# Genetic prediction of blood metabolites and causal relationships with pain associated with temporomandibular disorders: a Mendelian randomization study

**DOI:** 10.22514/jofph.2025.034

**Published:** 2025-06-12

**Authors:** Xue’e Zhang, Ketong Le, Weihong Xi

**Affiliations:** ^1^School of Stomatology, Jiangxi Medical College, Nanchang University, 330006 Nanchang, Jiangxi, China; ^2^Jiangxi Provincial Key Laboratory of Oral Diseases, 330006 Nanchang, Jiangxi, China; ^3^Jiangxi Provincial Clinical Research Center for Oral Diseases, 330006 Nanchang, Jiangxi, China

**Keywords:** Blood metabolites, Mendelian randomization, Temporomandibular joint pain, Causal relationships

## Abstract

**Background**: This study aims to elucidate the causal relationships between 1400 blood metabolites and pain related to temporomandibular disorders (TMD) using Mendelian Randomization (MR) analysis. **Methods**: Utilizing data from genome-wide association studies (GWAS), our analysis was conducted with R software using the “TwoSampleMR” package. The primary method applied was Inverse Variance Weighted (IVW) analysis, which was supplemented with MR-Egger, Weighted Median, Simple Mode and Weighted Mode methods to examine the causal impact of blood metabolites on TMD-associated pain. We also assessed heterogeneity and the presence of horizontal pleiotropy using MR-Egger regression, MR-PRESSO (MR Pleiotropy Residual Sum and Outlier) global tests, and MR-Egger intercept tests. **Results**: Three metabolites—Acetylcarnitine, Propionylcarnitine (c3) and X-24241—were significantly associated with TMD-related pain. Specifically, Acetylcarnitine had an odds ratio (OR) of 1.409 (95% CI (confidence intervals): 1.016–1.954, *p* = 0.04), Propionylcarnitine (c3) had an OR of 1.194 (95% CI: 1.036–1.377, *p* = 0.014), and X-24241 had an OR of 1.408 (95% CI: 1.108–1.790, *p* = 0.005). **Conclusions**: This study establishes a causal link between increased levels of these three metabolites and TMD-related pain. Our findings provide new insights into the pathogenesis of TMD and potential therapeutic targets.

## 1. Introduction

Temporomandibular disorders (TMD) constitute a heterogeneous group of conditions affecting the temporomandibular joints, masticatory muscles and related structures. Common symptoms include joint pain, abnormal jaw movements, clicking or popping sounds, and restricted mouth opening, making TMD a frequent concern in dental practice [1, 2]. Statistics show that the reported prevalence of TMD varies from country to country. The prevalence of TMD in the general U.S. population ranges from 10% to 26% [3], approximately 13.0% in Germany [4] and about 35% in Finland [5]. In a specific cohort of Chinese students, the prevalence is 29.1% [6]. Among affected individuals, pain associated with TMD is one of the primary symptoms and is the most common reason for seeking treatment, which captures a wide range of painful conditions associated with temporomandibular disorders [7]. A multicenter prospective cohort study found that 4% of individuals aged 18 to 44 without TMD are diagnosed with primary TMD-related pain annually, with new cases of TMD-related pain reaching 19% each year (defined as facial pain occurring for at least five days per month for one month or longer) [8]. This significantly impacts patients’ quality of life, yet treatment outcomes remain suboptimal due to the unclear etiology.

With recent breakthroughs in metabolomics technology, the identification of potential metabolic biomarkers and altered pathways has advanced our comprehension of disease mechanisms [9]. Some studies have shown that saliva metabolites may play an important role in pain, inflammation or joint disease [10]. Compared with salivary metabolites, blood metabolites are more widely distributed and stable in the body, so they have a wider range of applications in disease diagnosis, disease monitoring and treatment effect evaluation [11, 12]. Yet, the associative data provided by many metabolites often falls short of establishing causal links with diseases [13].

Mendelian randomization (MR), a robust method for causal inference, leverages genetic variants as instrumental variables (IVs) to deduce causal relationships between exposures and outcomes [14, 15]. In MR frameworks, genetic variations linked to the exposure of interest are sourced from genome-wide association studies (GWAS) and utilized in independent datasets to procure unbiased estimates of the relationships between exposures and outcomes [16]. MR approaches present distinct advantages over traditional epidemiological studies. Firstly, they minimize biases related to reverse causation [17]. Secondly, the random distribution of alleles during meiosis ensures that MR studies are not influenced by common behavioral, physiological, and socioeconomic confounders. Lastly, the structure of MR studies, similar to randomized controlled trials, alleviates ethical, feasibility and financial concerns significantly [18]. To date, studies have reported the use of MR to study the causal associations between sleep characteristics [19], educational level [20], autoimmune disorders [21], and psychiatric characteristics [22] with TMD.

However, MR has not been applied to explore the causal connections between blood metabolites and pain linked to TMD. This study utilizes MR analysis to examine the causal links between 1400 metabolites and pain associated with TMD, with the objective of identifying specific metabolites that directly contribute to TMD-related pain. The findings aim to shed light on targeted therapeutic approaches for managing temporomandibular pain, potentially paving way for more effective treatment strategies. This study follows the MR reporting guidelines outlined by STROBE-MR (Strengthening the reporting of observational studies in epidemiology using Mendelian randomization) to ensure transparency, reproducibility and quality in our research.

## 2. Materials and methods

### 2.1 Study design and data sources

The GWAS summary data used in this study were obtained from publicly available datasets and were formatted consistently. Essential information extracted related to the exposure factors included single nucleotide polymorphisms (SNPs), effect sizes, and the corresponding effect genes for each SNP. Other necessary information included the significance metrics (*p*-values) for the SNPs related to the exposure factors. The blood metabolites data were obtained from a publicly available GWAS meta-analysis, which includes 8299 individuals of European ancestry and comprises 1400 blood metabolites [23]. The TMD-related pain dataset was sourced from the FinnGen database, which includes 218,792 participants (4728 cases and 214,064 controls). For this study, we used ICD (International Statistical Classification of Diseases)-10 code K07.6 (“finn-b-DENTAL_TMD”) to identify cases of TMD-related pain. The summary statistics from this genome-wide association study have been archived in the GWAS Catalog, accessible via https://www.ebi.ac.uk/gwas/.

### 2.2 Selection of instrumental variables

To ensure the stability of the dataset and the accuracy of the results in this study where metabolites are used as exposures and TMD-related pain as the outcome, we established stringent criteria for selecting IVs: (a) In our study, we used a significance threshold of 1 × 10^−6^ for IVs linked to metabolites. This decision was made to mitigate the risk of false-negative findings that might arise from using an overly stringent threshold, such as 5 × 10^−8^, which could exclude SNPs with potential biological relevance. (b) We then performed linkage disequilibrium (LD) clustering with an *R*^2^ < 0.001 and an LD block length of 10,000 kb to reduce the effects of SNP correlation and guarantee the independence of genetic associations [24]; (c) The statistical robustness of the genetic variants used as IVs was evaluated using the *F*-statistic to mitigate biases in the causal inference between alleles, metabolites and TMD-related pain. IVs with an *F*-statistic ≤10 were considered weak and excluded due to their potential to produce biased outcomes, whereas an *F*-statistic >10 indicated strong IVs [25] (detailed information is available in **Supplementary Tables 1,2**). To quantify the relationship between the exposure variable and the outcome, we first calculate the proportion of variance explained by the exposure, denoted as *R*^2^. This is derived from the effect size and allele frequency of the exposure variable. The formula for *R*^2^ is expressed as follows:

$$R^{2}=\frac{2\times \beta_{\mathrm{exposure}}^{2}\times {\mathrm{eaf}}_{\mathrm{exposure}}\times (1-{\mathrm{eaf}}_{\mathrm{exposure}})}{2\times \beta_{\mathrm{exposure}}^{2}\times {\mathrm{eaf}}_{\mathrm{exposure}}\times (1-{\mathrm{eaf}}_{\mathrm{exposure}})+2\times {\mathrm{se}}_{\mathrm{exposure}}^{2}\times n_{\mathrm{exposure}}\times {\mathrm{eaf}}_{\mathrm{exposure}}\times (1-{\mathrm{eaf}}_{\mathrm{exposure}})}$$

Subsequently, to assess the significance of the model, we compute the *F* statistic, which relates the proportion of explained variance to the sample size. The formula for the *F* statistic is given by:

$$F=\frac{R^{2}\times (n_{\mathrm{exposure}}-2)}{1-R^{2}}$$

β_exposure_: Represents the effect size of the exposure variable (regression coefficient).

eaf_exposure_: Represents the effect allele frequency of the exposure variable.

se_exposure_: Represents the standard error of the effect of the exposure variable.

n_exposure_: Represents the sample size.

### 2.3 MR analysis

To explore the complex interactions between metabolites and pain associated with temporomandibular disorders, we methodically employed five distinct MR analytical techniques. These included Inverse Variance Weighted (IVW), MR-Egger, Weighted Median, Simple Mode and Weighted Mode [25, 26, 27, 28]. As our principal analytical strategy, the IVW method aggregates the Wald ratios of each IV, akin to a meta-analysis. The validity of our findings can be assessed by statistical efficacy calculations to assess whether each IV meets the MR assumptions, *post hoc* power calculations for MR analyses are available online via https://sb452.shinyapps.io/power/, and whether horizontal multidimensionality exists [29]. The MR-Egger regression is tailored to handle pleiotropic effects, asserting that these effects must be independent of the genetic exposure factors to yield precise causal estimates [27, 30]. If not all five methods reach the significance level of *p* < 0.05, we prioritize the IVW method. This is not only because a significant IVW result suggests a potential causal link between the exposure and the outcome, but also because the IVW method is a well-established and widely used statistical approach in MR studies. It combines information from multiple genetic variants, yielding more precise and robust estimates. Notably, if the IVW method indicates significant findings without evidence of pleiotropy or heterogeneity, and the effect direction is consistent across all analytical methods, we regard these results as robust—even if other methods do not reach statistical significance (*p* > 0.05).

To ensure the thoroughness of our findings, we conducted extensive sensitivity analyses. Initially, we assessed heterogeneity using Cochran’s Q and MR-Egger tests, where a *p*-value < 0.05 denotes significant heterogeneity. Furthermore, the MR-Egger intercept test was utilized to detect pleiotropy and verify the reliability of our results. A *p*-value < 0.05 in this test indicates the presence of pleiotropy. We also applied the MR Pleiotropy Residual Sum and Outlier (MR-PRESSO) method to meticulously check for pleiotropic bias. The findings were aligned with the IVW results after outlier exclusion [31]. Fig. 1 illustrates specific details of our study design.

**Fig. 1.** Conceptual framework of the MR study on the causal relationship between blood metabolites and pain associated with temporomandibular disorders. IV: Instrumental Variable; LD: Linkage Disequilibrium; MR: Mendelian Randomization; MR-PRESSO: MR Pleiotropy Residual Sum and Outlier; TMD: temporomandibular disorders.

### 2.4 Statistical analysis

To examine the causal links between the 1400 metabolites and pain associated with temporomandibular disorders, our study primarily utilized methods such as IVW and Weighted Median estimation. These analyses were conducted using the “TwoSampleMR” package, version 0.6.8, in the R software environment, which is specifically tailored for MR analyses and offers comprehensive tools for estimation, testing and sensitivity assessments of causal relationships [32]. The entire statistical procedure was carried out using R software, version 4.4.1, a premier platform for statistical computing and graphics, accessible at http://www.r-project.org/.

Data preparation and handling were meticulously performed to ensure the clarity and integrity of the findings. Odds ratios (OR) along with their 95% confidence intervals (CI) were systematically formatted for clear presentation. *p*-values were adjusted to improve the interpretability of the results. Furthermore, we addressed any missing values for exposure IDs and SNP counts meticulously, guaranteeing a robust dataset ready for detailed analysis.

## 3. Results

### 3.1 MR analysis

In this investigation, the IVW method served as the benchmark for evaluating the causal associations between blood metabolites and pain linked to TMD. We pinpointed three metabolites significantly associated with TMD-related pain: Acetylcarnitine (IVW: OR = 1.409, 95% CI: 1.016–1.954, *p* < 0.05), Propionylcarnitine (c3) (IVW: OR = 1.194, 95% CI: 1.036–1.377, *p* < 0.05), and X-24241 (IVW: OR = 1.408, 95% CI: 1.108–1.790, *p* < 0.05), as depicted in Fig. 2. The initial findings from the IVW analysis suggest that these metabolites act as risk factors for TMD-related pain, with increased levels correlating with a higher probability of experiencing this condition.

**Fig. 2.** Forest plot of the impact of metabolites on TMD-related pain (GCST90199669 refer to Acetylcarnitine; GCST90199708 refer to Propionylcarnitine (c3); GCST90200623 refer to X-24241). OR: Odds ratios; CI: confidence intervals; MR: Mendelian Randomization; val: value; nsnp: number of single nucleotide polymorphisms. Bold format represents *p* < 0.05 considered statistically different.

### 3.2 Reliability and stability analysis of results

In the IVW analysis, the Cochran’s Q test for Acetylcarnitine resulted in a *p*-value of 0.09, and the MR-Egger regression test showed a *p*-value of 0.10, both exceeding the threshold of 0.05, indicating the absence of heterogeneity in the study results. Similarly, both tests for Propionylcarnitine (c3) and X-24241 also yielded *p*-values greater than 0.05, further confirming no significant heterogeneity. Additionally, the Egger intercepts for these metabolites recorded *p*-values of 0.46, 0.84 and 0.78 respectively, all suggesting no horizontal pleiotropy. The comprehensive MR-PRESSO test results, with *p*-values of 0.32 for each metabolite, underscored the absence of significant outliers or deviations among the IVs, enhancing the robustness and validity of our MR findings (Table 1). The *post hoc* power calculations for the MR analysis were as follows: Power_Acetylcarnitine_ = 99.1%, Power_Propionylcarnitine(c3)_ = 83.8%, Power_X−24241_ = 97.8%.

**Table 1.** Sensitivity analysis of the impact of metabolites on TMD-related pain in MR study.

Exposure	Outcome	SNPs	Heterogeneity Test		Pleiotropy Test	MR-PRESSO
			MR-Egger Q (*p*)	Cochran’s Q (*p*)	Egger-intercept (*p*)	RSSobs (*p*)
Acetylcarnitine (GCST90199669)	TMD related pain	4	4.57 (0.10)	6.43 (0.09)	0.06 (0.46)	22.06 (0.32)
Propionylcarnitine (c3) (GCST90199708)	TMD related pain	6	6.68 (0.15)	6.76 (0.24)	0.01 (0.84)	13.50 (0.23)
X-24241 (GCST90200623)	TMD related pain	5	5.12 (0.16)	5.27 (0.26)	0.02 (0.78)	10.96 (0.29)

*TMD: temporomandibular disorders; SNPs: single nucleotide polymorphisms; MR: Mendelian Randomization; MR-PRESSO: MR Pleiotropy Residual Sum and Outlier; RSSobs: Residual Sum of Squares observed.*

The circular plot analysis, incorporating five distinct MR methods—IVW, MR-Egger regression, weighted median, simple mode, and weighted mode—highlighted blood metabolites associated with TMD-related pain. A smaller *p*-value in this context indicates a stronger association (Fig. 3).

**Fig. 3.** Circular plot illustrating the selection of blood metabolites associated with TMD-related pain, meeting the criteria of at least one of the five research methods. IVW: Inverse Variance Weighted; MR: Mendelian Randomization.

The forest plot’s horizontal axis represents effect sizes, with values greater than zero suggesting risk factors, and values less than zero indicating protective factors. The combined effect sizes of the associated SNPs were positive, identifying Acetylcarnitine, Propionylcarnitine (c3) and X-24241 as risk factors for TMD-related pain (Fig. 4).

**Fig. 4.** Forest plots showing composite effects of SNPs related to metabolites. (A) Forest plot showing the cumulative effect of SNPs associated with Acetylcarnitine on TMD-related pain; (B) Forest plot illustrating the cumulative effect of SNPs associated with Propionylcarnitine (c3) on TMD-related pain; (C) Forest plot depicting the cumulative effect of SNPs associated with X-24241 on TMD-related pain. MR: Mendelian Randomization.

Leave-one-out analysis for these metabolites showed that the overall effect estimates lay to the right of the null line, indicating that no single SNP dominated the observed associations. Stepwise exclusion analysis revealed no significant impact of any individual SNP on the causal estimates (Fig. 5).

**Fig. 5.** Leave-one-out analysis forest plots for metabolites. (A) Forest map of the analysis of metabolite Acetylcarnitine; (B) Forest map of the analysis of metabolite Propionylcarnitine (c3); (C) Forest map of the analysis of metabolite X-24241. MR: Mendelian Randomization.

The scatter plot used five algorithms to regress metabolite SNPs, displaying SNP effects on metabolites along the x-axis and their impact on TMD-related pain along the y-axis. The upward trajectory of the lines indicates that as metabolite levels increase, so does the risk of developing TMD-related pain. This confirms a significant positive causal relationship between Acetylcarnitine, Propionylcarnitine (c3) and X-24241 and TMD-related pain, with demonstrated stability of the included SNPs (Fig. 6).

**Fig. 6.** Scatter plots showing the risk of pain associated with TMD related to metabolites. (A) Scatter plot of the risk associated with Acetylcarnitine for TMD-related pain; (B) Scatter plot of the risk associated with Propionylcarnitine (c3) for TMD-related pain; (C) Scatter plot of the risk associated with X-24241 for TMD-related pain. MR: Mendelian Randomization; SNP: single nucleotide polymorphisms.

## 4. Discussion

TMD are among the four most common dental conditions, characterized by complex etiologies and often uncertain treatment efficacy, presenting significant challenges in clinical practice. The diagnostic criteria for temporomandibular disorders (DC/TMD) categorize TMD into two major categories: (1) joint disorders, which include disc dislocations, degenerative conditions, and intra-articular inflammatory conditions, and (2) painful disorders, which encompass muscle pain, joint pain and TMD-related headaches [33, 34]. TMD-related pain not only severely affects patients’ quality of life but can also impact their mental health [35]. The predominant clinical treatment strategies focus mainly on symptomatic relief to alleviate pain, largely due to a limited understanding of the underlying mechanisms of TMD-related pain. TMD pain symptoms are believed to result from complex interactions among physiological, psychological and social factors [2, 36]. Research has identified significant roles for large molecular substances such as pain mediators (*e.g.*, substance P, prostaglandins, thromboxane) [37] and inflammatory factors (*e.g.*, interleukins IL-1, IL-2, IL-6) [38, 39]. Additionally, increased expression of matrix metalloproteinases (MMP-1, MMP-2 and MMP-13) has been linked to joint inflammation and the progression of pain [40]. While changes in blood metabolites are suspected to influence the onset of TMD-related pain, the genetic basis of these metabolites remains underexplored.

This study, through MR analysis, identified three metabolites—Acetylcarnitine, Propionylcarnitine (c3) and X-24241—that show a positive causal relationship with the occurrence of TMD-related pain, indicating that elevated levels of these metabolites increase the risk of developing this condition. Acetylcarnitine plays a key role in transporting long-chain fatty acids across mitochondrial membranes, making it an essential molecule for energy metabolism [41]. In addition to its primary metabolic functions, Acetylcarnitine has antioxidant properties that protect against oxidative stress. It regulates neurotransmitters through epigenetic mechanisms and has shown neurotrophic and analgesic activity in experimental models of chronic inflammation and neuropathic pain [42, 43, 44]. Studies have reported lower concentrations of acetylcarnitine in patients with osteoarthritis compared to healthy individuals [45], suggesting that Acetylcarnitine could be a valuable biomarker for musculoskeletal disorders, including TMD-related pain. Our study shows that alterations in Acetylcarnitine levels may be linked to TMD-related pain, warranting further research to explore whether modulating Acetylcarnitine could be a potential therapeutic target. Propionylcarnitine (c3) is significant in metabolic conditions like propionic acidemia and methylmalonic acidemia [46]. Recent evidence suggests that fluctuations in propionylcarnitine levels are correlated with chronic pain phenotypes, including those related to inflammatory responses [47]. Our study highlights the potential role of propionylcarnitine in modulating the inflammatory processes associated with TMD-related pain, making it a potential marker for identifying and managing chronic pain in TMD patients. An intriguing finding was the discovery of an “unknown” metabolite, X-24241, which, although less well studied, was hypothesized to be potentially involved in TMD-related pain based on its structural similarity to metabolites related to pain modulation and oxidative stress. Although its chemical nature remains undefined, the identification of a causal relationship with TMD-related pain suggests that further studies of X-24241 may yield valuable insights. This suggests that acetylcarnitine, propionylcarnitine and X-24241 may serve as measurable indicators of metabolic dysregulation in patients with TMD. Understanding the mechanism of action of these metabolites in TMD-related pain can help develop targeted therapeutic strategies. For example, based on the changes in these metabolic markers, we can develop personalized prevention plans, such as modifying dietary habits and increasing the intake of specific nutrients, to prevent the occurrence of TMD or promote recovery. Furthermore, it is necessary to conduct more rigorous studies, including longitudinal studies and clinical trials, to validate the roles of these metabolites and explore their broader applications in clinical practice.

Despite these advancements, this study’s limitations must be acknowledged. The datasets may include unknown confounding factors influencing the outcomes, for example, there may be selectivity bias in selecting the FinnGen database for analysis. Moreover, the GWAS summary data, primarily derived from European populations, may not be universally applicable. The lack of individual metabolite level data prevents a detailed analysis of how specific metabolites contribute to different types of pain (*e.g.*, muscle pain, joint pain, TMD headaches). The definition of TMD-related pain in this study covers a wide range of pain symptoms associated with TMJ disorders, which ensures inclusiveness of all TMD-related pain types; however, more refined subcategories could provide additional insights for future studies. Therefore, DC/TMD-specific diagnoses need to be included in future studies to improve clinical relevance. Moreover, while the study suggests causal relationships, it does not explore the precise mechanisms by which metabolites influence TMD-related pain. In conclusion, this study underscores the potential of molecular mechanisms in exploring the causal relationships between blood metabolites and TMD-related pain, providing a robust theoretical foundation for future clinical research. However, further studies are needed to validate these findings and elucidate the mechanisms involved, enhancing the potential for targeted treatment strategies.

## 5. Conclusions

In summary, this study explored the causal relationship between blood metabolites and temporomandibular disorder (TMD)-related pain using Mendelian randomization. Our findings elucidate the risk factors and potential protective factors associated with TMD-related pain, emphasizing the complex interplay of genetic and metabolic factors in its pathogenesis. However, a comprehensive understanding of the underlying mechanisms by which these metabolites influence TMD-related pain is crucial for developing targeted therapeutic strategies. At the same time, it is extremely important to validate these causal relationships through experimental and longitudinal studies, especially in different populations and pain classifications. These studies will not only provide deeper insights to reveal new therapeutic targets for the prevention and treatment of TMD-related pain, but they are also expected to change the outcome of dental medicine for patients.

## Supplementary Material

Supplementary material associated with this article can be found, in the online version, at https://files.jofph.com/files/article/1933045171506561024/attachment/Supplementary%20material.xlsx.


